# A Case of Aspergillus calidoustus Thoracic Spine Osteomyelitis

**DOI:** 10.7759/cureus.65667

**Published:** 2024-07-29

**Authors:** Chidi D Okoroafor, Madhu Suryadevara, Parveen Gaba, Polly Jen

**Affiliations:** 1 Infectious Diseases, Newark Beth Israel Medical Center, Newark, USA; 2 Internal Medicine, Trinity Health of New England, New Haven, USA; 3 Pharmacy, Newark Beth Israel Medical Center, Newark, USA

**Keywords:** transplant infections, fungal spine infections, aspergillus calidoustus, spinal osteomyelitis, fungal infections

## Abstract

*Aspergillus* infections are of significant concern in patients who are immunocompromised, including transplant recipients*. Aspergillus calidoustus* is an emerging pathogen reported to cause a wide array of infections. We present a case of *A. calidoustus* thoracic spine osteomyelitis in a patient with an orthotopic heart transplant (OHT). To our knowledge, this is the first case of *A. calidoustus* osteomyelitis in a patient with OHT.

## Introduction

*Aspergillus calidoustus* is a mold pathogen within the group *ustus* [1]. It has been gaining prominence as an emerging pathogen due to the increased use of antifungal prophylaxis in transplant patients and the resultant emergence of more resistant *Aspergillus* species. Solid organ transplant (SOT) and hematopoietic transplant (HCT) patients receiving immunosuppressive therapy tend to be at the greatest risk, particularly those receiving anti-mold azole prophylaxis, such as voriconazole or posaconazole [2]. Infections attributed to *A. calidoustus *may involve the lungs, central nervous system, or soft tissues, either as isolated or disseminated infections [2,3]. *Calidoustus* infections have been found to be increasingly resistant to antifungals, mostly triazoles, in contrast to other *Aspergillus* species, with the minimum inhibitory concentrations (MICs) of voriconazole of some *Aspergillus* species 2-16 mcg/mL and posaconazole MICs of ≥1-16 mcg/mL [2]. We present a case of *A. calidoustus *osteomyelitis affecting the thoracic spine in a patient who is one year post OHT.

## Case presentation

A 64-year-old gentleman presented to the emergency room with a month-long history of sharp mid and lower back pain that had progressively worsened since the onset. He was prescribed cyclobenzaprine a few weeks prior to presentation with no significant improvement in his symptoms. He denied fever, bowel or bladder dysfunction, and upper and lower extremity weakness, but endorsed tingling of his left shoulder and left arm. The patient was afebrile on arrival with a blood pressure of 128/85 mmHg and a respiratory rate of 18 breaths per minute. Physical examination was significant for left arm paresthesia.

Significant past medical history

Orthotopic heart transplant for chronic heart failure and ischemic cardiomyopathy was performed one year ago for which he was placed on valganciclovir for CMV prophylaxis, Bactrim for antibacterial prophylaxis, and voriconazole for antifungal prophylaxis. The patient's current immunosuppression regimen consists of tacrolimus and mycophenolate mofetil.

The patient has a significant history of pulmonary aspergillosis diagnosed by pathology following a lung biopsy 13 months ago for which the patient was initially treated with voriconazole and micafungin and then switched to isavuconazole with voriconazole discontinued due to side effects.

Magnetic resonance imaging (MRI) of the spine with and without contrast done on admission was significant for ventral epidural abscess of T9-T10 with discitis and osteomyelitis, moderate spinal canal stenosis, and ventral cord impingement. Due to these findings, infectious disease and neurosurgery services were consulted. The patient was taken to the operating room by the neurosurgery service where a T9-T10 laminectomy was done with attempted abscess evacuation; however, no evidence of frank pus was noted during surgery. No growth was noted on intraoperative bacterial cultures. Fungal and acid-fast bacilli cultures were not collected. The surgical pathology of the T9-T10 specimen sent was consistent with fibrous tissue and blood clots.

The patient was empirically started on cefepime and daptomycin for suspected bacterial vertebral osteomyelitis. On the 23rd day of admission, repeat MRI imaging of the thoracic spine showed persistence of T9-T10 changes with discitis and osteomyelitis. Computed tomography (CT)-guided biopsy of the T9-T10 thoracic spine was done by interventional radiology on the 27th day of hospitalization with the pathology report consistent with prior findings. On the 40th day of hospitalization, the patient underwent thoracic spine decompression with arthrodesis done by neurosurgery with bone cultures sent for microbiology. Bone culture preliminary report was significant for rare fungus. Liposomal amphotericin B was initiated on day 42 based on the preliminary results. The bone culture specimen was sent to a reference laboratory at the University of Texas at San Antonio for further identification and susceptibility testing. The final bone culture report was positive for *A. calidoustus* with susceptibility results indicating amphotericin B MIC = 0.5 mcg/mL, micafungin MIC ≤ 0.015 mcg/mL, rezafungin MIC ≤ 0.015 mcg/mL, voriconazole MIC 4 mcg/mL, isavuconazole MIC 1 mcg/mL, and posaconazole MIC 4 mcg/mL. The decision was made to treat *Aspergillus* vertebral osteomyelitis with prolonged combination therapy, so micafungin was added to liposomal amphotericin B following the final culture report. The patient was discharged on day 82 of hospitalization and was clinically stable with upper and lower extremity motor and sensory function still preserved.

**Figure 1:**
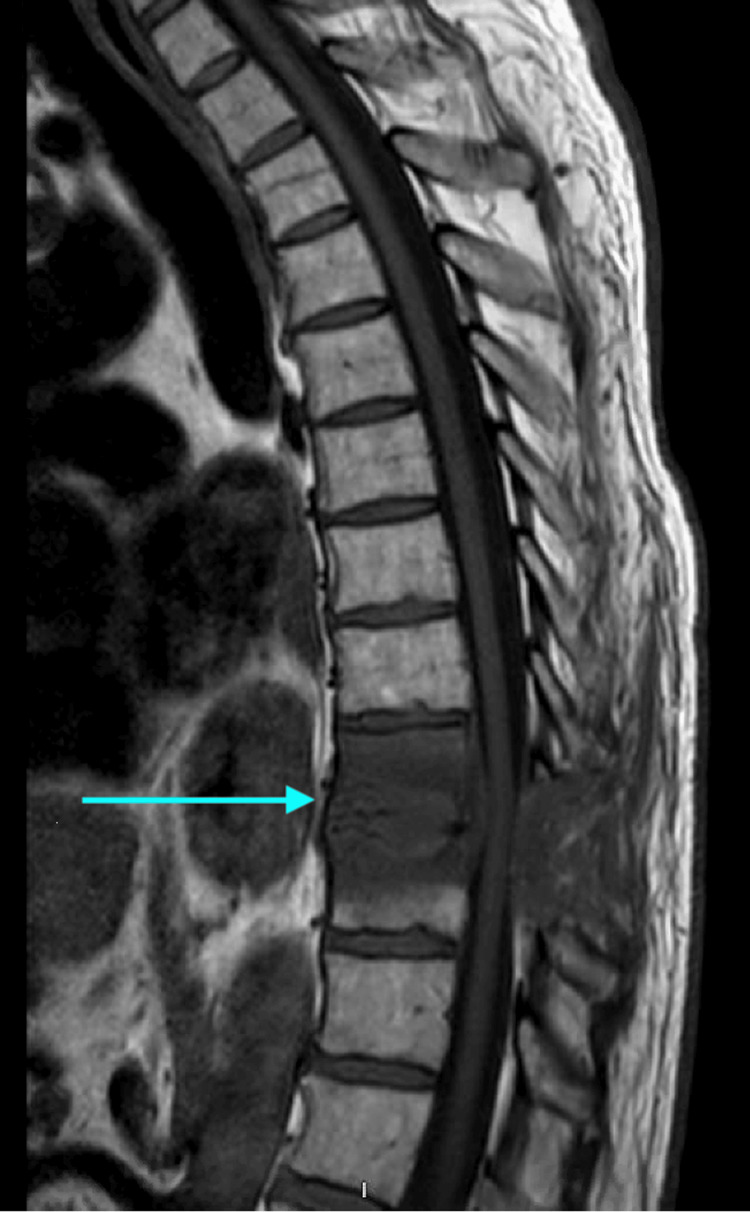
MRI of the thoracic spine with the T9-T10 area being involved.

**Figure 2:**
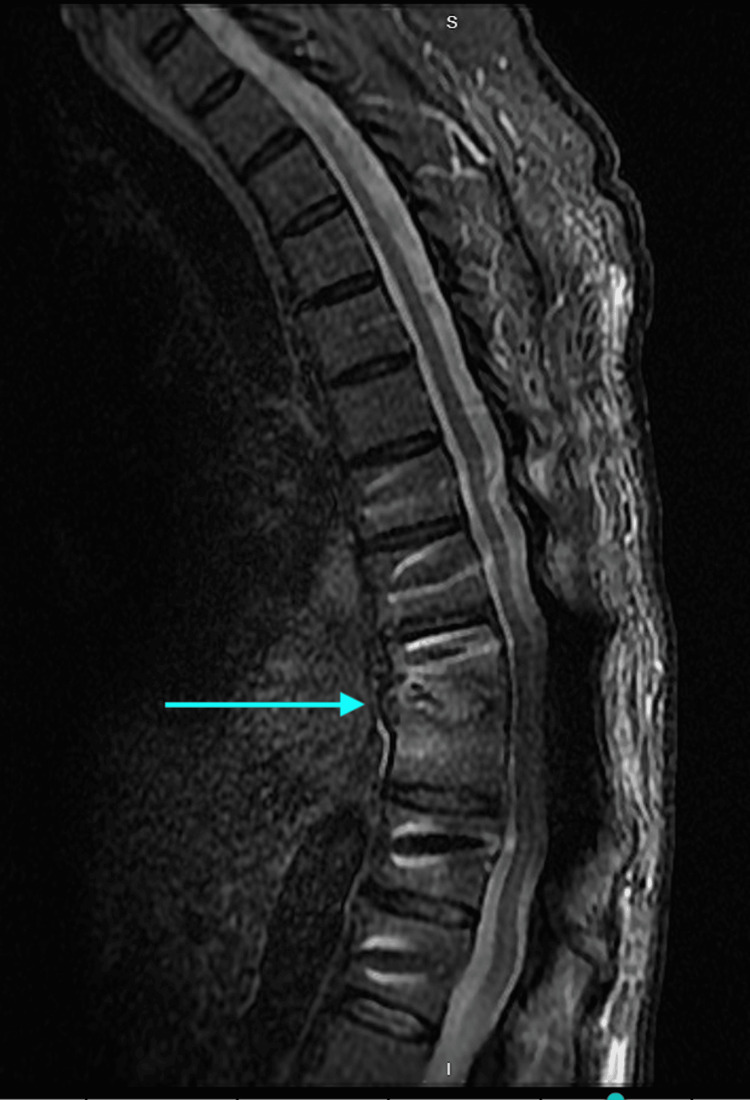
MRI of the thoracic spine with the T9-T10 area now with significant improvement in the lesion.

MRI findings pre- and post-initiation of antifungal treatment with liposomal amphotericin B and micafungin are shown in Figure 1 and Figure 2, with significant improvement in the imaging of vertebral lesions eight weeks into treatment compared to when it was initiated.

## Discussion

*A. calidoustus *is a recently defined species of *Aspergillus* reported as a growing cause of infections in immunosuppressed individuals. While over 300 *Aspergillus* species are known to exist, including many others in the same section of *Usti*, *A. calidoustus* is distinct as a thermotolerant mold that is able to grow at 37 °C, similar to the human body temperature [1]. In addition, the appearance of *A. calidoustus* differs from the closely related *A. ustus*, where the mycelium color on Czapek yeast extract agar for *A. calidoustus* is grayish yellow to grayish brown compared to the cream or light-yellow appearance of *A. ustus *[2,3].

*A. calidoustus *is commonly isolated from food, soil, and air; however, infections due to this species have been increasingly reported. The relative prevalence of *A. calidoustus* causing infections among *Aspergillus* species has been reported to be 1.4% in Spain and 2.8% in the United States [2,4-6]. Similar to other *Aspergillus* infections, solid organ and hematopoietic transplant patients are at increased risk for infection due to the need for immunosuppressive medications to prevent graft rejection [2]. However, the treatment of *A. calidoustus *infections is notoriously difficult due to the high MICs for most antifungals, with decreased susceptibility to azoles attributed to the prevalence of prophylaxis use in transplant patients [1-2]. The reported susceptibility patterns for *A. calidoustus* have been variable with a wide range of MICs reported: voriconazole 2-16 μg/mL, posaconazole ≥1-16 μg/mL, caspofungin 0.03-32 μg/mL [2], and amphotericin B 0.25-32 μg/mL [2,4-10]. The in vitro susceptibility results from our case are consistent with those previously published as seen in this case series [2]. While susceptibility breakpoints have not been established by professional organizations for *A. calidoustus s*pecies, the triazole MICs reflect the need for concentrations that exceed those that can be reasonably achieved using standard doses of triazole antifungals based on their pharmacokinetic and pharmacodynamics properties. The use of non-azole antifungals, as a monotherapy or combination therapy, has been reported with success in isolated cases or case series [2,10,11].

There is very limited published experience on *A. calidoustus* infections in transplant patients. One case series [2] described a 67-year-old female with a history of lung transplantation who developed thoracic back pain and was found to have enhancement of the T12-L1 disc space and leptomeningeal enhancement of the anterior surface of the spinal cord at the T12 level. Image-guided intervertebral disc aspiration was performed, and a fungal culture grew one colony of *A. calidoustus* [2]. One of the other cases in the cases series describes *A. calidoustus*-related pulmonary aspergillosis, brain abscesses, and maxillary and ethmoidal sinus infection (in the same patient) secondary to *A. calidoustus*, all in HCT patients on immunosuppression. To the authors’ knowledge, there are no cases in the literature describing heart transplant patients with infections secondary *A. calidoustus* infections as described in this case.

In transplant recipients, infections caused by *A. calidoustus* have been found to have mostly occurred within the first few months or years following receipt of SOT or HCT, although some episodes occurred many years after transplantation [2]. As discussed in this case series from the TRANSNET database, the median onset of infection caused by all *Aspergillus* species among HCT recipients was noted to be 99 days [2]. It has been discussed in the literature that in SOT recipients in the United States, the median time to invasive aspergillosis (IA) caused by any species was 184 days post SOT [2], with a wide interquartile range of approximately one month to 27 months post SOT [2]. Approximately 20% of IA cases occurred more than three years after transplantation. The patient in our case appears to have developed a disseminated infection with the index organ being the lungs as he was diagnosed and treated for pulmonary aspergillosis over a year ago. At that time, the diagnosis was based upon the pathology of the lung tissue from biopsy as no isolate was sent for culture. *A. calidoustus* infections in SOT patients have been treated with a combination of antifungal therapy and surgery as in our case [1-2,10]. Confirmatory data are lacking with respect to clinical efficacy and achievement of cure using this approach. Furthermore, the incidence of toxicities associated with antifungal therapy, as a single or combination therapy, was not well-described. The potential risks and benefits of combination antifungal therapy versus surgery must be considered by clinicians when making treatment decisions in patients with these infections.

## Conclusions

This is a unique case of thoracic spine osteomyelitis caused by *A. calidoustus* in a heart transplant recipient, from possible dissemination after an index pulmonary infection. As an emerging pathogen, it is important to understand the epidemiology and pathogenesis of this organism in humans. This report represents the first described case of vertebral osteomyelitis in a heart transplant patient due to *A. calidoustus*.

This case also highlights the predisposition of transplant patients on immunosuppression developing resistant fungal infections as in this case, due to the prior use of prophylactic antifungal agents post transplant.
